# Effect of computerized cognitive remediation therapy on mental time travel in patients with schizophrenia— a pilot randomized controlled trial

**DOI:** 10.3389/fpsyt.2024.1363290

**Published:** 2024-05-02

**Authors:** Junhua Cao, Cao Zhou

**Affiliations:** ^1^ School of Medicine, Henan Polytechnic University, Jiaozuo, Henan, China; ^2^ Department of Psychiatry, The Second People’s Hospital of Guizhou, Guiyang, Guizhou, China

**Keywords:** schizophrenia, computer cognitive remediation therapy, mental time travel, remember the past, imagine the future

## Abstract

**Objective:**

To investigate the intervention effect of computerized cognitive remediation therapy (CCRT) on mental time travel (MTT) in patients with schizophrenia(SCZ).

**Methods:**

From August 2020 to July 2021, 60 patients with SCZ were randomly allocated to either the study or the control group. The control group was treated with conventional drugs alone. The study group received CCRT and medical therapy for 40 minutes three times a week for 4 weeks. The participants underwent the MTT test before and after the training.

**Results:**

A total of 28 patients in the study group and 26 patients in the control group were included in the analysis. Before training, there was no significant difference in the concretization ratio of recalling past and imagining future events between the study group and the control group (P > 0.05). After 4 weeks of training, the specific event ratio of the study group was higher than that of the control group (P < 0.01). In terms of the emotional titer of the events, the concreteness of the positive events in the study group was higher than that of the neutral events and negative events (P < 0.01). The concreteness of negative events was higher than that of neutral events (P < 0.01).

**Conclusion:**

CCRT can improve the MTT ability of SCZ patients, which is manifested by an increase in the concretiveness of recalling past and imagining future events.

## 1 Introduction

Schizophrenia(SCZ) is a chronic severe mental illness with a worldwide prevalence of approximately 1% (1). Patients suffer from attention disorder, memory disorder, executive dysfunction and other cognitive impairment, including mental time travel (MTT) (2). MTT refers to an individual’s ability to recall the past or imagine the future. The ability to mentally relive past events is called a mental time travel pointing to the past (recalling the past), while the ability to mentally pre-experience future events is called a mental time travel pointing to the future (imagining the future). Projecting oneself into the past (recalling the past, i.e., autobiographical memory) is closely related to projecting oneself into the future (wanting to go to the future) (3). MTT plays an important role in our daily life. For example, it can help people achieve goals, cope with stress, and make decisions (4). Deficits in MTT affect the ability to recall of specific events and imagine the future, which may lead to problems such as impaired problem-solving and decreased overall functionality (5, 6). Therefore, the study of MTT in SCZ has important clinical significance.

Existing studies on MTT indicate that there is a deficit in MTT in patients with SCZ, which is manifested in issues in remembering the past and imagining the future. In terms of remembering the past, people with SCZ recall fewer events and lack details (7). Regarding the future, people with SCZ have difficulties in imagining it in detail (8), and the deficits are more pronounced than those related to remembering the past (9). Presently, researchers are exploring ways to improve MTT ability, one of which is cognitive correction training. Computerized cognitive remediation therapy (CCRT) is a brain-training method used in the study of SCZ and affective disorders using a series of targeted tasks to enhance learning and improve patients’ cognitive abilities (10). Over the past 20 years, the number of trials investigating the efficacy of CCRT in SCZ spectrum disorder has increased significantly (11), and there is evidence that CCRT improves cognitive function in patients with SCZ, and that the benefits persist long after treatment has ended, particularly in memory, attention, and executive functioning (12).

Two recent meta-analyses examined the CCRT approach. One study found that CCRT had a small to moderate effect on attention, working memory, positive symptoms, and depressive symptoms (13). The second study evaluated the effects of CCRT on cognition, function, and clinical outcomes in patients with SCZ in 67 studies and found that CCRT treatment had significant improvements in small to moderate effects in all three areas (14).

So far, the current research mainly focuses on cognitive function, and there are few reports on mental time travel of patients with SCZ. The purpose of this study was to evaluate the efficacy of CCRT on MTT in patients with SCZ. Our main hypothesis is that CCRT will improve MTT ability in patients with SCZ.

## 2 Materials and methods

### 2.1 Materials

#### 2.1.1 Study design

This was a longitudinal, randomized, single-blind trial conducted at the Second People’s Hospital of Guizhou Province and Guiyang Lindong Hospital (the Intensive Medical Association of the Second People’s Hospital of Guizhou Province). This study has been approved by the ethical review of the Second People’s Hospital of Guizhou Province with the number [2020-SEYWYH-017]. All subjects gave informed consent to the study and signed informed consent form before starting the training.

#### 2.1.2 Participants

**Figure 1:**
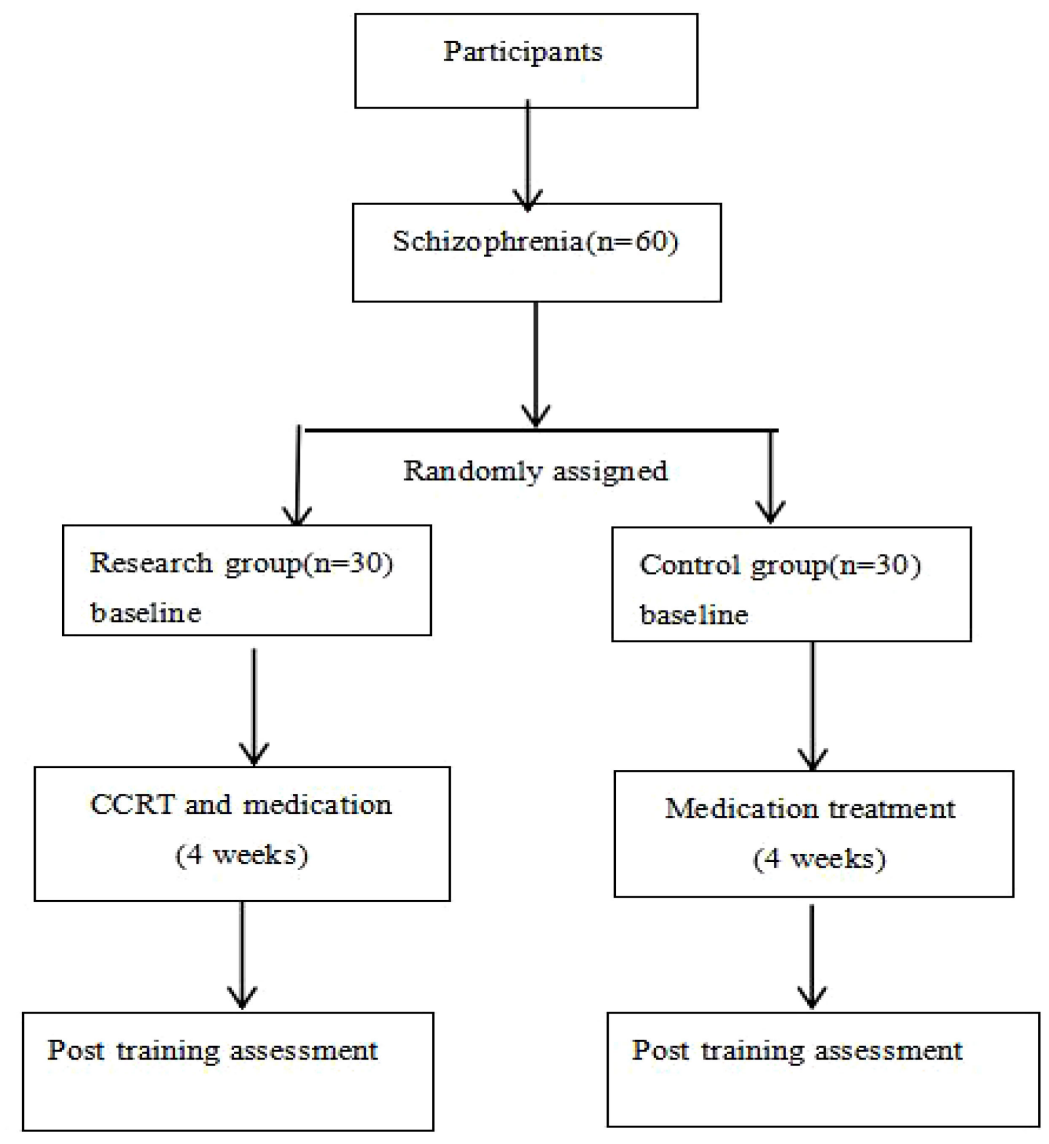
Flow chart of the study design.

From August 2020 to July 2021, 60 inpatients diagnosed with SCZ were recruited from the Second People’s Hospital of Guizhou Province and Guiyang Lindong Hospital. Inclusion criteria were (1) SCZ that meets the diagnostic criteria of the International Classification of Diseases-10^th^ Revision (ICD-10), with the Positive and Negative Syndrome Scale (PANSS) score not exceeding 60 points; (2) no history of neurological diseases; (3) no history of drug or alcohol dependence; (4) no electroconvulsive therapy within the past three months; (5) age of 18–50 years; (6) patients primary or higher education; (7) the intelligence quotient (IQ) of ≥70. Exclusion criteria comprised (1) mental retardation; (2) organic mental illness; (3) no cooperation due to declined or impulsive excitement; (4) severe anxiety, depression, or substance abuse; (5) auditory or visual perception impairment; (6) physical function diseases or other drug adverse reactions, not allowing patients to conduct computer game training in time; (7) pregnant or lactating women. In the end, 2 refused to participate, 4 were dislodged with a change in condition, and a total of 54 patients participated in the study. Experimental flow is shown in **Figure 1**.

#### 2.1.3 Randomization and blinding

Participants were randomly assigned 1:1 (the study group and control group), and randomization was conducted independently by psychiatrists not involved in the study after completing all baseline assessments. A table of random numbers was used to generate randomization, and the two groups were balanced in terms of age, sex, education, and disease course, as would be expected from random assignment. The patients and research personnel who are responsible for data collection, end-point evaluation, and statistical analysis will be blinded to the allocation of the two intervention arms. However, it is impractical to blind the attending physicians due to the nature of the treatment. Therefore, clinicians and researchers will not be blinded to the treatment allocation.

### 2.2 Methods

#### 2.2.1 Tools

##### 2.2.1.1 Sociodemographic data

We collected patients’ basic information, including age, sex, name, years of education, course of disease, medication dosage, etc.

##### 2.2.1.2 MTT measurement

The MTT measurement adopted in this study was adapted from the Autobiographical Memory Test(AMT)developed by Williams et al (15). The AMT consists of 10 different validities (5 positive cue words, 5 negative cue words). Subjects were asked to name a specific event related to the cue word within one minute. The criteria for concreteness event were: a specific time and place, and an event lasting no more than 1 day. Emotional titer is a dimension of emotion, which is divided into positive, neutral and negative. The emotional titer of events includes positive events, negative events and neutral events. The MTT measurement consists of two main parts: remembering the past and imagining the future (3). In this test, participants were asked to remember specific events in the past or imagine possible events in the future based on cue words, For example, the following question could be asked: “Can you describe a specific event or scene that has or has not occurred before but may occur in the future and is related to the cue word (e.g., a garden).” According to Ozdes et al. this paper has 15 cue words for recall and imagery respectively, including 5 each for positive, neutral and negative (4). For each cue word, participants had 1 minute to think and then describe the event. The Participants’ responses were recorded and then transcribed into words to judge the concretiousness of the described events and classify them into: (1) specific events, recalled or imagined events that occurred within a specific time of day; (2) Extensibility events, events lasting longer than one day;(3) A class event is a class of things that may occur frequently; (4) A semantically relevant description, which is not a thing, but may be an expression of a state or feeling; (5) No answer or can’t think of anything. The composition ratio of specific events to all described events was calculated as the main index of this test, and the composition ratio of recalled specific events and future specific events was divided according to the time direction, and the composition ratio of positive, neutral and negative specific events was divided according to the emotional titer of cue words. In order to ensure the reliability of the score, two raters scored the data of some participants at the same time, and the consistency coefficient of the raters was 0.85, indicating good consistency.

##### 2.2.1.3 Mini-international neuro-psychiatric interview

Studies have shown that MINI has good reliability as well as high inter-investigator agreement, and has been widely used in multicenter clinical drug studies and clinical practice (16). Patients with SCZ and their psychotic symptoms were diagnosed and controlled, respectively, by MINI with attending psychiatrists. The reliability and validity of neuropsychiatric interviews were good.

##### 2.2.1.4 Revised Wechsler Adult Intelligence Scale in China

IQ is an estimate of adult intelligence (17), which was assessed using the Chinese-revised Wechsler Adult Intelligence Scale based on four main subtests, including general knowledge, arithmetic, similarity, and number breadth. First, the rough score of each subtest was measured and then converted into the scale score. Then, the scale score of the four subtests was added, followed by dividing the total number by 4 and multiplying by 11. Finally, the corresponding IQ value was queried according to the norm table of different ages.

##### 2.2.1.5 Verbal fluency test

A verbal fluency test was used to detect speech priming (18). The subjects had 60 seconds to name the animal that came to mind. If the name of the animal was repeated, no score would be given. The number of correct animal names was the main index, which was positively correlated with the score.

##### 2.2.1.6 Alphanumeric span test

The alphanumeric span test was used to test working memory (19). Trained doctors read out combinations of characters (e.g., A, B, C, butyl, pente, and heptyl) and numbers (e.g., 123456789). The subjects were asked to first rank the numbers from the smallest to the largest and then answer the characters according to “A, B, C, butyl, pente, and heptyl.” The correct number was the main detection index.

##### 2.2.1.7 PANSS

The PANSS was used to assess the severity of psychiatric symptoms. PANSS has seven standards: 1 — none, 2 — very light, 3 — mild, 4 — moderate, 5 — heavy, 6 — severe, and 7 — extremely severe (20).

#### 2.2.2 Intervention methods

The control group was treated with conventional drugs for 4 weeks. The study group received CCRT and 40 minutes of training 3 times a week, 12 times for 4 weeks, in addition to conventional drug therapy. It is supervised by an experienced therapist with a ratio of 1:4 participants. The therapist taught the participants to use CCRT for the first two weeks, and the subsequent treatment was mostly done by the participants alone. CCRT equipment is provided by Beijing Dixin Technology Co., LTD, and involved cognitive flexibility, working memory, and planning to perform three therapeutic tasks. Each treatment task consisted of 6–10 different cognitive correction exercises, and each cognitive correction training program consisted of 10–30 training tasks of varying difficulty. Specific content included continuous matching, quick matching, finding differences, shopping planning, picture classification, and emotion management. Before the initial treatment, the computer will evaluate the subjects and give the training plan. The subjects will undergo cognitive training under the guidance of the therapist. In each training, the content of 3 modules will appear, and the computer will automatically match the corresponding progress and difficulty according to the subjects’ achievements in the training module. Special staff will call to remind subjects in advance on the day of treatment, and those who complete the 4-week training will be rewarded to improve their compliance.

#### 2.2.3 Sample size

According to relevant studies, this paper conducted technical tests on the sample size selection. Specifically, the effect value was set as 1, the significance level as 0.05, and the statistical testing force as 0.9. The minimum sample size was calculated as 23 for each group through G*power software, and the sample size of each group was finally determined to be 30 people considering the loss rate of 10%.

#### 2.2.4 Statistical methods

SPSS 22.0 was used for data analysis. A t-test and *χ^2^* test were used to compare the two groups of general data. 2 (group: study group, control group) × 2 (time point: before intervention, after intervention) × 2 (time direction: memory, imagination) × 3 (emotional titer: positive, neutral, and negative) repeated measures of ANOVA were performed, and η² was used to represent the effect size, the greater the value, the greater the degree of difference. Test level *α* = 0.05.

## 3 Results

### 3.1 Demographic and clinical information

A total of 60 patients with SCZ participated in the study: 30 in the study group and 30 in the control group. Within 4 weeks of treatment, one patient in the MTT group was excluded due to discharge, another one was excluded due to aggravation of the disease, two patients in the control group were excluded due to inability to adhere to the evaluation, and two patients were excluded due to recurrence of the disease. Finally, 28 patients in the study group and 26 patients in the control group could complete the training after enrollment. Both groups were given risperidone, olanzapine, aripiprazole, and other drugs, which were converted into equivalent doses of chlorpromazine. Antipsychotic dose, general psychopathological symptoms, positive symptoms, negative symptoms, education level, sex, age, and IQ were not significantly different between the two groups (**Table 1**).

**Table 1:** Demographic and clinical information of participants.

Project	Study (n = 28)	Control (n = 26)	*t/χ^2^*	*P*
Male/Female	14:14	14:12	0.074^a^	0.785
Age(years)	37.43 ± 8.48	41.58 ± 8.70	−1.775^b^	0.082
IQ	79.16 ± 5.96	78.90 ± 9.49	0.120^b^	0.905
Education(years)	9.54 ± 3.32	9.69 ± 3.80	−0.162^b^	0.872
Duration of illness (years)	11.38 ± 7.30	11.29 ± 6.93	0.045^b^	0.965
General psychopathology	26.39 ± 2.38	26.39 ± 2.17	0.013^b^	0.989
Positive symptoms	8.50 ± 1.29	8.15 ± 1.85	0.803^b^	0.426
Negative symptoms	14.50 ± 2.53	15.58 ± 2.77	−1.492^b^	0.142
PANSS	49.39 ± 4.21	50.12 ± 3.64	−0.672^b^	0.505
Medication (CPZ mg/d)	191.51 ± 119.09	220.00 ± 149.63	−0.777^b^	0.441

^a^represents the value of χ^2^, and ^b^represents the value of t.

### 3.2 Comparison of MTT concreteness between the two groups before and after intervention

The concrete descriptive results of MTT in the two groups before and after the intervention are shown in **Table 2**. In the two groups, the task to remember past and imagine future specific events was repeated by 2 (group: study group, control group) × 2 (time point: before intervention, after intervention) × 2 (time direction: remember, imagination) × 3 (emotional titration: positive, neutral, and negative). The results showed that the main effect was significant [F(1, 52) = 24.76, P < 0.01, η² = 0.323], and the specific event ratio in the study group was higher than that in the control group. The time point main effect was significant [F(1, 52) = 139.21, P < 0.01, η² = 0.728], and the specificity of the test after training was higher than that before training. The time direction main effect was significant [F(1, 52) = 24.20, P < 0.01, η² = 0.318], and the specificity of remembering past events was higher than that of imagining the future. The main effect of emotional titer was significant [F(1, 52) = 127.57, P < 0.01, η² = 0.833]. Further analysis showed that the concreteness of positive cue words was higher than that of neutral and negative cue words, and that of negative cue words was higher than that of neutral words (P < 0.01). Group and the interaction between before and after the test significantly [F (1, 52) = 85.56, P < 0.01, eta squared = 0.622]. Simple effect analysis showed that there was no significant difference between the two groups before training, and the concreteness of the study group was significantly higher than that of the control group after CCRT training (P < 0.01).

**Table 2:** Descriptive statistical results of mental time travel concreteness in both groups after 4 weeks of intervention (x ± s).

Point in time	Emotional titer	Study (n = 28)		Control (n = 26)	
		pre-training	post-exercise	pre-training	post-exercise
remember	positive	0.11 ± 0.07	0.28 ± 0.13	0.13 ± 0.06	0.15 ± 0.11
	neutral	0.03 ± 0.05	0.08 ± 0.10	0.02 ± 0.04	0.02 ± 0.03
	negativity	0.06 ± 0.07	0.12 ± 0.10	0.06 ± 0.05	0.07 ± 0.07
imagine	positive	0.09 ± 0.07	0.33 ± 0.09	0.09 ± 0.06	0.12 ± 0.09
	neutral	0.02 ± 0.03	0.03 ± 0.05	0.02 ± 0.03	0.01 ± 0.03
	negativity	0.05 ± 0.07	0.10 ± 0.01	0.03 ± 0.04	0.06 ± 0.07

## 4 Discussion

The mental time travel ability of schizophrenic patients is defective (21), affecting cognitive, emotional, and behavioral processes (22). It is difficult to recall the past and imagine the future, which makes it difficult for people to make decisions, solve problems, plan for the future, and make reasonable time estimates for activities of daily living. At present, some scholars have begun to explore the methods that can improve mental time travel ability, including life review training, autobiographical memory training and cognitive training (23, 24). Although life review therapy and autobiographical memory training were used in patients with SCZ, the above-mentioned studies have limitation. Due to the obvious decline in memory in some patients, there is resistance to life review training and autobiographical memory training, and most patients have poor compliance. This study adopted CCRT therapy, making full use of the advantages of computer technology in task standardization, material enrichment, objective evaluation, difficulty adjustment, and other aspects, to maximize the adaptability and compliance of treatment.

After four weeks of CCRT treatment, The results show that CCRT has a certain effect on the concreteness of past and future events in patients with SCZ. This is similar to Blairy’s findings (25), which found that cognitive training improved the ability to recall specific events, and cognitive correction therapy was mentioned as an effective intervention to improve autobiographical memory. Therefore, this study extends on the basis of Blairy’s research. In addition, Garrido and Linke found that CCRT can improve the attention, memory, executive function and other cognitive fields of SCZ patients (26, 27). CCRT improves working memory, attention and executive function and problem-solving abilities of patients with SCZ and thus improve the ability to recall the past and imagine the future (28, 29).

This study shows that CCRT can be an effective intervention for the MTT of patients with SCH. This provides preliminary evidence for the role of CCRT in MTT in patients with SCZ. CCRT is a behavioral training method that has been shown to have a relatively good therapeutic effect on improving cognitive deficits in SCZ patients (30). Compared with other methods that can be used to improve cognitive impairment in patients with SCZ, CCRT has advantages such as high patient acceptance, simple operation, low safety risks, extensive data and easy access. Therefore, as a new therapy, CCRT has become one of the cognitive rehabilitation intervention tools for SCZ patients at home and abroad.

## 5 Strengths and limitations

Regarding the strengths of this study, We are the first to use the CCRT method to improve the effect of patients with schizophrenia on the MTT. There are still some shortcomings in this study, which should be further explored in future studies. The limitations of this study are as follows. First, the clinical training period of this study was relatively short, and the long-term effect was not tracked. Future studies can extend the training time to further explore the effects of CCRT on MTT, cognition, and clinical symptoms of patients with SCZ. Second, no follow-up evaluation was conducted, and future clinical studies should include follow-up evaluation as much as possible, such as three months and six months after the completion of training. Finally, the health study group was not included. Whether the treatment for CCRT is likely to improve the ability to remember the past and imagine the future to a healthy level needs to be further explored. Future research directions can include the health group for comparison.

## 6 Conclusion

CCRT can provide an effective, simple and economical way to improve SCZ. In the future, it could be integrated into the overall treatment of people with mental illness, which could be a key part of recovery and relapse prevention for people with mental illness.

## Data availability statement

The original contributions presented in the study are included in the article/supplementary material. Further inquiries can be directed to the corresponding authors.

## Ethics statement

This study was approved by the ethics review of the Second People’s Hospital of Guizhou Province with the number. The studies were conducted in accordance with the local legislation and institutional requirements. The participants provided their written informed consent to participate in this study.

## Author contributions

JC: Investigation, Data curation, Writing – review & editing, Writing – original draft. CZ: Writing – review & editing, Project administration, Methodology, Funding acquisition.
